# Extracellular circulating viral microRNAs: current knowledge and perspectives

**DOI:** 10.3389/fgene.2013.00120

**Published:** 2013-06-24

**Authors:** Alessandro Laganà, Francesco Russo, Dario Veneziano, Sebastiano Di Bella, Rosalba Giugno, Alfredo Pulvirenti, Carlo M. Croce, Alfredo Ferro

**Affiliations:** ^1^Department of Molecular Virology, Immunology and Medical Genetics, Comprehensive Cancer Center, The Ohio State UniversityColumbus, OH, USA; ^2^Department of Clinical and Molecular Biomedicine, University of CataniaCatania, Italy; ^3^Laboratory for Integrative System Medicine, Institute of Informatics andTelematics and Institute of Clinical Physiology, National Research CouncilPisa, Italy

**Keywords:** microRNA, viruses, exosomes, circulating microRNA, vesicules, body fluids

## Abstract

MicroRNAs (miRNAs) are small non-coding RNAs responsible of post-transcriptional regulation of gene expression through interaction with messenger RNAs (mRNAs). They are involved in important biological processes and are often dysregulated in a variety of diseases, including cancer and infections. Viruses also encode their own sets of miRNAs, which they use to control the expression of either the host’s genes and/or their own. In the past few years evidence of the presence of cellular miRNAs in extracellular human body fluids such as serum, plasma, saliva, and urine has accumulated. They have been found either cofractionate with the Argonaute2 protein or in membrane-bound vesicles such as exosomes. Although little is known about the role of circulating miRNAs, it has been demonstrated that miRNAs secreted by virus-infected cells are transferred to and act in uninfected recipient cells. In this work we summarize the current knowledge on viral circulating miRNAs and provide a few examples of computational prediction of their function.

## INTRODUCTION

MicroRNAs (miRNAs) are the most studied and best characterized molecules in the class of small regulatory non-coding RNAs (Bartel, 2009). They are involved in several important biological processes and functions through post-transcriptional regulation of the expression of messenger RNAs (mRNAs), and their dysregulation is often cause or consequence of a variety of diseases, such as cancer and neurodegenerative disorders (Croce, 2009; Eacker et al., 2009). Cellular miRNAs can be packaged into different carriers and exported to recipient cells or released in small vesicles during apoptosis (Boon and Vickers, 2013; Hilton and Karpe, 2013). The discovery of extracellular miRNAs in biological fluids has started a new exciting field of research. Circulating miRNAs are now considered useful markers of disease conditions and functional mediators of several biological processes in a novel form of cell-to-cell communication. A novel useful resource is the database miRandola, which provides users with a comprehensive manually curated classification of extracellular circulating miRNAs (Russo et al., 2012).

Viruses encode their own sets of miRNAs. Evidence shows that these miRNAs can act as self-regulators of viral gene expression and/or control host cell pathways through silencing of their nodes (Kincaid and Sullivan, 2012). Some viruses can exploit extracellular particles for the initiation and progression of the infection and recent evidence indicates that viruses can export and deliver functional miRNAs through vesicles (Pegtel et al., 2010). This discovery reveals a new layer in the infectious mechanism used by viruses to maintain their latency and control crucial host pathways whose targeting is likely beneficial to the virus.

In this mini review we summarize the current knowledge about circulating miRNAs and their potential regulatory functions, with particular emphasis on extracellular viral miRNAs. We report the promising results of the most recent studies and provide a few examples of computational prediction of viral miRNA function.

## CURRENT KNOWLEDGE

### CIRCULATING miRNAs ARE FUNCTIONAL IN RECIPIENT CELLS AND CONSTITUTE USEFUL BIOMARKERS FOR VARIOUS CONDITIONS

Extracellular miRNAs have been recently identified stable in most biological fluids, including blood, urine, saliva, semen, cerebrospinal fluid, and breast milk (Mitchell et al., 2008; Hanke et al., 2010; Wang et al., 2011; Alexandrov et al., 2012; Gallo et al., 2012; Zhou et al., 2012). Evidence shows that they may be selectively packaged into different kinds of carriers, such as membrane-derived vesicles, lipoproteins, and ribonucleoprotein complexes, which protect them from degradation and export them to recipient cells where they exert their regulatory functions. Particularly, exosomes and microparticles (MPs) are two distinct classes of small membrane-enclosed vesicles released from cells, differing in size, biogenesis, and secretory mechanisms (Boon and Vickers, 2013). Exosomes are produced by the inward budding of the limiting membrane of multivesicular bodies (MVBs). They are smaller than MPs, which are instead formed by the outward budding and blebbing of the plasma membrane. Small sealed membrane vesicles that are produced from cells during apoptosis, called apoptotic bodies, can also transport specific sets of miRNAs. Extracellular miRNAs have also been found in high-density lipoproteins (HDL) and low-density lipoproteins (LDL), and bound to Argonaute 2 (AGO2) and other ribonucleoproteins, both in and out of membrane-derived vesicles (Arroyo et al., 2011; Turchinovich et al., 2011; Vickers and Remaley, 2012; Rayner and Hennessy, 2013). Viral surface antigen particles may also carry specific miRNAs, as in the case of hepatitis B surface antigen particles which contain hepatocellular miRNAs bound to AGO2 (Novellino et al., 2012).

MicroRNA profiles of extracellular carriers show distinct sets of miRNAs than their parent cell-type, thus suggesting that some miRNAs might be transcribed only to be exported and not retained in the parent cell (Ohshima et al., 2010; Pigati et al., 2010). Selective packaging of miRNAs into vesicles is probably related to the specific biological functions of the secreted miRNAs.

Circulating miRNAs are highly stable and consistent among individuals of the same species. Specific miRNA expression signatures in extracellular environment have been identified in a variety of human diseases, including cancer and neurological diseases, revealing the diagnostic potential of circulating miRNAs as useful non-invasive biomarkers (Alexandrov et al., 2012; Fayyad-Kazan et al., 2013; Zeng et al., 2013).

Several *in vitro* studies have shown that miRNAs transferred by the different types of carriers are functional and can regulate gene expression in recipient cells.

Apoptotic bodies generated from endothelial cells during atherosclerosis were shown to contain miR-126, which controls endothelial cell signaling *in vitro* and provides atheroprotective effects *in vivo* (Zernecke et al., 2009).

Another study showed that endothelial cells can transfer functional miR-143 and miR-145 to smooth muscle cells where they mediate the reduction of atherosclerotic lesion formation *in vivo* (Hergenreider et al., 2012).

Similarly, circulating miR-150 is released by monocytes and taken up by endothelial cells where it regulates endothelial cell migration (Zhang et al., 2010).

Although the complete mechanism of gene regulation mediated by specifically selected extracellular circulating miRNAs has yet to be clearly demonstrated *in vivo*, these studies suggest a plausible form of cell-to-cell communication in which donor cells send their miRNAs to distant recipient cells where they exert their regulatory functions.

### VIRUSES EXPLOIT EXTRACELLULAR PARTICLES TO ESTABLISH AND MAINTAIN THE INFECTION

It has been shown that some viruses exploit extracellular particles, such as microvesicles, for the initiation and progression of the infection (Meckes and Raab-Traub, 2011). According to the trojan exosome hypothesis proposed by Gould et al. (2003), retroviruses may use the pre-existing non-viral exosome biogenesis and uptake pathways for the formation, release, and delivery of viral particles.

This has been later supported by evidence that some viruses utilize endosomal compartments of the host to generate exosome-like vesicles (Hosseini et al., 2013) which can play different roles in the infection, contributing to its spreading (Mack et al., 2000), favoring exosomal biogenesis (daSilva et al., 2009), and providing immune evasion (Temme et al., 2010).

Viral exosomes, for instance, affect the host immune system in different ways according to the type of virus and the stage of its life cycle in which exosome secretion occurs in the infected host. As proving example, during the non-replicative stage, dendritic cells serve as transit location for HIV-1 (human immunodeficiency virus 1) which exploits their intracellular vesicle trafficking pathways to release antigens and viral particles into the extracellular space and *trans*-infect CD4+ T cells (Izquierdo-Useros et al., 2010).

Generally, viruses implement different strategies during infection essentially consisting in escaping the host immune system and facilitating the invasion and proliferation within the host. Observations suggest that the release of microvesicles containing specific cellular and viral components by infected cells contributes greatly to the preservation of the virus even in a hostile antiviral immune environment (de Gassart et al., 2003; Izquierdo-Useros et al., 2009; Klibi et al., 2009; György et al., 2011; Meckes and Raab-Traub, 2011).

Epstein–Barr virus (EBV), cytomegalovirus (CMV), and hepatitis C virus (HCV) have found means to evade immune responses and increase virus-fusing ability and infectivity by exploiting microvesicles, giving rise to a systematic distribution of viral agents from infected cells able to induce genetic and epigenetic modifications in recipient cells (Masciopinto et al., 2004; Klibi et al., 2009; Plazolles et al., 2011; Wurdinger et al., 2012).

Tumor-associated viruses, like EBV, may use exosomal transfer to manipulate the growth characteristics of neighboring cells and enhance tumor progression. In particular, exosomes released from nasopharingeal carcinoma (NPC) cells harboring latent EBV were shown to contain the EBV latent membrane protein 1 (LMP1; Meckes et al., 2010), which is frequently expressed in EBV-associated cancers and has potent effects on cell growth by inducing growth-stimulating signaling pathways (Wang et al., 1985; Kaye et al., 1993) and may modulate the selective sorting of proteins into exosomes, favoring important signaling molecules frequently activated in cancers such as phosphatidylinositol 3-kinase (PI3K) and epidermal growth factor receptor (EGFR; Meckes et al., 2010).

### VIRUSES ENCODE miRNAs

RNA interference (RNAi) most probably was originally selected as a primary mechanism of defense against harmful genetic elements such as viruses. It is of relevant interest that in the evolutionary selection this mechanism was in turn exploited by viruses to their advantage while, as suggested by tenOever (2013), chordate use of small RNAs might exclusively have shifted to the silencing of genome-encoded transcripts and would at least not pose direct threat to RNA viral genome.

The first report of viral-encoded miRNAs was published by Pfeffer et al. (2004) describing the cloning of viral miRNAs from cells infected with EBV. Among DNA viruses, which account for the majority of known virus-encoded miRNAs, 95% of viral miRNAs known today are of herpesvirus origin.

The majority of natural viruses found to encode miRNAs have thus a DNA component to their replication cycle, can exploit the initiating host miRNA biogenesis machinery in the nucleus where they replicate, and cause long-term persistent infections. DNA viruses such as the ones belonging to the Herpesvirus, Polyomavirus, Ascovirus, Baculovirus, Iridovirus, and Adenovirus families clearly match these characteristics (Sullivan et al., 2005; Gottwein et al., 2007; Choy et al., 2008; Hussain et al., 2008; Seo et al., 2009; Seto et al., 2010; Bauman et al., 2011; Marquitz et al., 2011; Suffert et al., 2011; Zhao et al., 2011; Lee et al., 2012) along with at least one member of the retrovirus family, bovine leukemia virus (BLV), which clearly encodes numerous miRNAs (Kincaid et al., 2012).

Despite the established case of BLV, viruses possessing positive or negative sense RNA or double-stranded RNA (dsRNA) genome are not widely accepted to naturally express miRNAs.

Nevertheless, HIV-1 has been proven to encode two miRNAs and potentially a third. In fact, hiv1-mir-H1 was proven to be responsible for inducing apoptosis and repressing host gene expression (Kaul et al., 2009), while hiv-1-miR-N367 has been suggested as functional ortholog of hsa-miR192 (You et al., 2012). Finally, some evidence is present that the HIV-1 TAR element could be a potential viral miRNA (Houzet and Jeang, 2011), also considering its capability to target pro-apoptotic genes (Klase et al., 2009).

All viral miRNAs can essentially be grouped into two classes: host analogs and virus-specific. Generally, though, their functions include prolonging longevity of infected cells, evading the immune response, and regulating host or viral genes to limit the lytic cycle. Interestingly, all these functions are essential for infections to be persistent.

In fact, miRNAs are likely invisible to the adaptive immune system – a valuable trait for viruses that undergo persistent infection (Cullen, 2006). Thus, in viruses that establish a long-lasting latent infection, such as herpesviruses, one important benefit they could gain from employing miRNAs is the ability to regulate host and/or viral gene expression without having to elicit an antigenic immune reaction or directly suppressing components of the host immune system (Sullivan, 2008).

Preventing cell death seems an obvious advantage to viruses that cause persistent or latent infections. Several different viruses including Kaposi’s sarcoma-associated herpesvirus (KSHV), EBV, and Marek’s Disease Virus type 1 (MDV1) encode miRNAs that can play a subtle role in preventing apoptosis by targeting pro-apoptotic host genes and are also associated with tumorigenesis.

## PERSPECTIVES

### VIRUSES CAN USE VESICLES TO EXPORT THEIR FUNCTIONAL miRNAs

Pegtel et al. (2010) were the first ones (and, to our knowledge, the only ones together with Meckes et al., 2010) to have demonstrated that virus-infected cells package virus-encoded RNAs, and specifically viral miRNAs, into exosomes which are exported into the extracellular space and eventually delivered to recipient, non-infected cells, favoring the repression of specifically important mRNA targets. EBV is a clear example of a virus that utilizes the exosome pathway for the selective secretion of viral and cellular proteins and miRNAs that likely participate in cell-to-cell communication in the absence of virus production, potentially modulating cell function.

As confirming proof, Pegtel et al. (2010) reported that EBV-infected activated B cells secrete exosomes containing viral miRNAs shown to be delivered and actively internalized by monocyte-derived dendritic cells in co-culture. In particular, the copy number of EBV-miRNA BART1-5p was consistently higher than other EBV-miRNAs and its level increased fourfold after additional 24 h co-culture. This resulted in a dose-dependent, miRNA-mediated repression of confirmed EBV target genes. More specifically, the viral miRNA BHRF1-3 was shown to suppress the expression of the immunostimulatory gene CXCL11 [Chemokine (C-X-C motif) ligand 11] and this repression was proven to be dependent on the amount of exosomes carrying the miRNA and was not recipient cell-type-specific. In addition, expression of EBV-miRNAs in EBV-infected circulating B cells was also investigated. The data collected suggested that in asymptomatic patients BART miRNAs are expressed by latently infected circulating B cells as well as present in non-infected non-B cells, supporting the possibility of miRNA transfer *in vivo*. This further supported the proposal that exosomes could most likely serve as deliverers of small RNA due to their specialized biogenesis and presumed entry route (Zomer et al., 2010).

Later evidence showed that EBV-encoded miRNAs have been detected in exosomes from EBV-infected NPC cells, together with the LMP1 protein and other signal transduction molecules (Meckes et al., 2010), in accordance to other studies proving the presence of cellular miRNAs in tumor-derived exosomes (Taylor and Gercel-Taylor, 2008; Kharaziha et al., 2012; Palma et al., 2012).

Furthermore, differences detected in the levels of intracellular and exosomial miRNAs, in addition to differences even in the amount of enrichment between the individual exosomal miRNAs, suggest that some viral miRNAs might be specifically intended and selected to be packaged into exosomes and exert their functions in cells other than those producing them (Klibi et al., 2009; Meckes et al., 2010; Pegtel et al., 2010). Moreover, exosomes may also deliver cellular components of the RNA-induced silencing complex (RISC) to enhance viral miRNA function (Gibbings et al., 2009).

These results were greatly motivated by the assumption that exosomal exportation of miRNAs in general may have a fundamental role in intercellular communication despite the lack of concrete evidence (Valadi et al., 2007; Skog et al., 2008; Théry et al., 2009).

**FIGURE 1:**
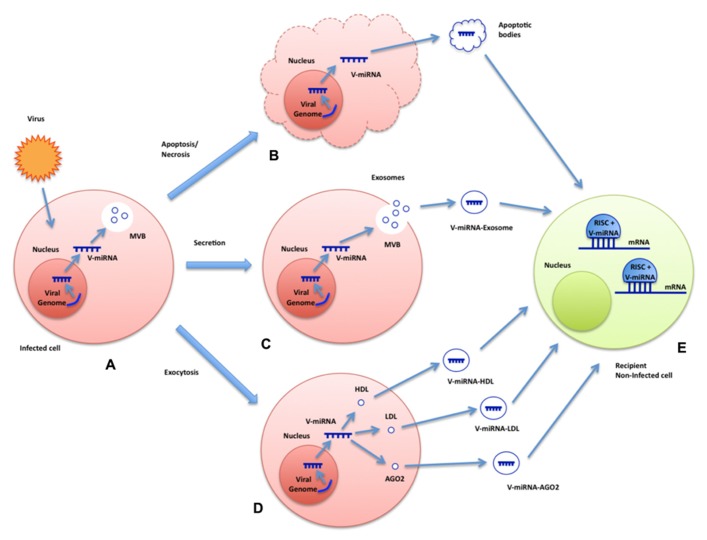
**Summary model of plausible mechanisms for export and functional delivery of viral miRNAs.** The image depicts the possible means of transcription, packaging, and functional delivery of viral miRNAs during an infection. Virus-encoded miRNAs are transcribed by the infected cell **(A)**. They could exploit various channels to reach extracellular space and, eventually, be delivered to recipient non-infected cells: inside apoptotic bodies after cell death **(B)**, packaged into exosomes **(C)**, or HDL/LDL molecules or even bound to AGO2 **(D)**. Viral miRNAs may be uptaken by non-infected cells where they could exert their regulatory functions **(E)**.

Although functional significance of all these phenomena requires further investigation, these results suggest that a cellular miRNA-loading mechanism may exist to direct specific miRNAs into intraluminal vesicles of multivesicular endosomes (MVEs) which could explain why exogenous exosomal miRNAs are capable of repressing targets in recipient cells at new subcellular compartments for RNAi activity such as late endosomes (Morelli et al., 2004; Stern-Ginossar et al., 2007; Gibbings et al., 2009). **Figure 1** depicts all the potential ways in which viruses could exploit extracellular particles to convey their miRNAs to non-infected recipient cells.

### FUNCTIONAL ANALYSIS OF CIRCULATING VIRAL miRNAs

The correct identification of targets is fundamental to determine miRNA function. Computational miRNA target prediction is still a big challenge, mostly due to the fact that our knowledge about the mechanisms and the molecular rules of miRNA target recognition is still incomplete (Bartel, 2009). Nevertheless, there are many computational tools available online, which allow to identify the most probable miRNA targets and to uncover non-trivial relationships between miRNAs and other molecular actors (Cascione et al., 2013). These tools collect and integrate heterogeneous miRNA-related data retrieved from different sources, such as target prediction tools and expression profiles of miRNAs and mRNAs, in order to infer miRNA functions and produce general models of miRNA-mediated regulation in the context of complex processes. Few tools are available specifically for the analysis of viral miRNAs and they are limited to the prediction of new miRNAs and targets. RepTar and vHoT are databases of predicted interspecies interactions between viral miRNA and host genomes, while ViTa is a database containing predictions of host miRNA targets on viruses (Hsu et al., 2007; Elefant et al., 2011; Kim et al., 2012). miRiam is a software that has been used to predict potential human targets for viral miRNAs (Laganà et al., 2010). Finally, VMir and Vir-Mir are tools for the prediction of novel virus-encoded miRNAs (Li et al., 2008; Grundhoff, 2011). In regard to functional analysis, despite the lack of specific programs for viral miRNAs, general miRNA tools can be successfully applied to the study of viral miRNAs as well. A very recent study shows that the predicted targets of the 135 known viral miRNAs in human viruses and of 6809 putative miRNAs encoded by 23 human viruses, as predicted by Vir-Mir, are enriched for specific host pathways whose targeting is likely beneficial to the virus, such as cancer, axon guidance, ErbB, mitogen-activated protein kinase (MAPK), and wingless-type MMTV integration site family (Wnt) signaling (Carl et al., 2013). The authors performed a functional enrichment analysis by comparing each gene target set with an annotated functional gene set corresponding to KEGG (Kyoto Encyclopedia of Genes and Genomes) pathways and Gene Ontology biological processes. As further proof of principle, we used miRiam to predict the potential targets of EBV miRNAs in which exosomes are particularly enriched, as reported by Pegtel et al. (2010) (miR-BHRF1-1/1-2-3p and miR-BART1-3p/5p/-2-3p). Then, we used the tool ingenuity pathway analysis (IPA) to perform a functional enrichment analysis of the predicted targets (). The results show that subsets of the targets are significantly involved in cancer pathways, in particular leiomyomatosis, and mesenchymal tumors, for which a connection with EBV had already been described (Cheuk et al., 2002; Monforte-Muñoz et al., 2003; Deyrup et al., 2006; Sunde et al., 2010). Other significant pathways include WNT/B-catenin signaling, interleukin 8 (IL-8) signaling, and P53 pathway (*P* < 0.0001), also previously described as related to EBV infections (Morrison et al., 2003; Everly et al., 2004; Ren et al., 2004; Webb et al., 2008; Forte and Luftig, 2009; Husaini et al., 2011; QingLing et al., 2011). The predicted targets are also enriched in GO terms such as cell death and survival and cell cycle (*P* < 0.04). Furthermore, although the significance of the *P*-value is borderline (*P* < 0.4), it is worth to mention that the top tox functions reported by IPA include increased levels of alkaline phosphatase and LDH, tumour-marker characteristics which have been reported to be significant prognostic factors in metastatic NPC, often associated wih EBV infection (Jin et al., 2012). **Table 1** summarizes the most significant associations.

**Table 1:** Functional enrichment analysis of circulating EBV miRNAs’ predicted targets.

	*P*-Value
**Selected canonical pathways**
Molecular mechanisms of cancer	5.27 × 10^-^^11^
PPARα/RXRα activation	9.39 × 10^-^^6^
Wnt/β-catenin signaling	1.25 × 10^-^^5^
p53 signaling	6.73 × 10^-^^5^
IL-8 signaling	1.56 × 10^-^^4^
**Selected molecular and cellular functions**
Cell morphology	<3.6 × 10^-^^2^
Cell death and survival	<3.72 × 10^-^^2^
Cell cycle	<4.09 × 10^-^^2^
**Selected diseases and disorders: cancer**
Leiomyomatosis	1.21 × 10^-^^5^
Cell transformation	2.60 × 10^-^^3^
Growth of tumor	5.14 × 10^-^^3^
Mesenchymal tumor	8.31 × 10^-^^3^
**Selected tox functions (Clinical Chemistry and Hematology)**
Decreased levels of albumin	1.37 × 10^-^^1^
Increased levels of alkaline phosphatase	2.21 × 10^-^^1^
Increased levels of albumin	3.70 × 10^-^^1^
Increased levels of LDH	3.70 × 10^-^^1^

*The table summarizes the most relevant results, particularly associated to EBV infection, of the functional analysis conducted using the software IPA. Results are organized in categories. For each category, the most significant terms, together with their *P*-Values, are displayed. EBV-encoded miRNAs in which exosomes are particularly enriched were selected (miR-BHRF1-1/1-2-3p and miR-BART1-3p/5p/-2-3p) and their targets predicted using the tool miRiam. The top scoring targets were given as input to IPA.*

These few examples clearly indicate that miRNA functional analysis tools can be of great help in studying the effects of circulating viral miRNAs, allowing the production of plausible hypotheses about their function and involvement in crucial cellular pathways, encouraging the development of more specific tools for computational investigation of cellular and extracellular viral miRNA.

## Conflict of Interest Statement

The authors declare that the research was conducted in the absence of any commercial or financial relationships that could be construed as a potential conflict of interest.
